# In Cellulo Examination of a Beta-Alpha Hybrid Construct of Beta-Hexosaminidase A Subunits, Reported to Interact with the GM2 Activator Protein and Hydrolyze GM2 Ganglioside

**DOI:** 10.1371/journal.pone.0057908

**Published:** 2013-03-04

**Authors:** Incilay Sinici, Sayuri Yonekawa, Ilona Tkachyova, Steven J. Gray, R. Jude Samulski, Warren Wakarchuk, Brian L. Mark, Don J. Mahuran

**Affiliations:** 1 Department of Biochemistry, Hacettepe University, Faculty of Medicine, Ankara, Turkey; 2 Genetics and Genome Biology, Research Institute, Hospital for Sick Children, Toronto, Ontario, Canada; 3 Department of Laboratory Medicine and Pathobiology, University of Toronto, Toronto, Ontario, Canada; 4 Gene Therapy Center, University of North Carolina, Chapel Hill, North Carolina, United States of America; 5 Ryerson University, Department of Chemistry and Biology, Toronto, Canada; 6 Department of Microbiology, University of Manitoba, Winnipeg, Manitoba, Canada; University Hospital S. Maria della Misericordia, Italy

## Abstract

The hydrolysis in lysosomes of GM2 ganglioside to GM3 ganglioside requires the correct synthesis, intracellular assembly and transport of three separate gene products; i.e., the alpha and beta subunits of heterodimeric beta-hexosaminidase A, E.C. # 3.2.1.52 (encoded by the *HEXA* and *HEXB* genes, respectively), and the GM2-activator protein (GM2AP, encoded by the *GM2A* gene). Mutations in any one of these genes can result in one of three neurodegenerative diseases collectively known as GM2 gangliosidosis (*HEXA*, Tay-Sachs disease, MIM # 272800; *HEXB*, Sandhoff disease, MIM # 268800; and *GM2A*, AB-variant form, MIM # 272750). Elements of both of the hexosaminidase A subunits are needed to productively interact with the GM2 ganglioside-GM2AP complex in the lysosome. Some of these elements have been predicted from the crystal structures of hexosaminidase and the activator. Recently a hybrid of the two subunits has been constructed and reported to be capable of forming homodimers that can perform this reaction in vivo, which could greatly simplify vector-mediated gene transfer approaches for Tay-Sachs or Sandhoff diseases. A cDNA encoding a hybrid hexosaminidase subunit capable of dimerizing and hydrolyzing GM2 ganglioside could be incorporated into a single vector, whereas packaging both subunits of hexosaminidase A into vectors, such as adeno-associated virus, would be impractical due to size constraints. In this report we examine the previously published hybrid construct (H1) and a new more extensive hybrid (H2), with our documented in cellulo (live cell- based) assay utilizing a fluorescent GM2 ganglioside derivative. Unfortunately when Tay-Sachs cells were transfected with either the H1 or H2 hybrid construct and then were fed the GM2 derivative, no significant increase in its turnover was detected. In vitro assays with the isolated H1 or H2 homodimers confirmed that neither was capable of human GM2AP-dependent hydrolysis of GM2 ganglioside.

## Introduction

There are two major lysosomal beta-hexosaminidase (Hex) isozymes in the normal human tissue; i.e., the highly stable Hex B, a homodimer of beta subunits (encoded by the *HEXB* gene) and the less stable Hex A, a heterodimer composed of beta and alpha (encoded by the *HEXA* gene) subunits. These genes are evolutionarily related with the primary structures of the two subunits they encode being ∼60% identical. Whereas Hex B and Hex A share many of the same natural substrates, only Hex A can hydrolyze the non-reducing terminal, beta-linked, N-acetyl galactosamine residue from the acidic glycolipid GM2 ganglioside (GM2) to produce GM3 ganglioside. Because the hydrophobic GM2 normally resides in a membranous environment, Hex A is sterically hindered from efficiently binding it in vivo. This problem is overcome by the presence of a small lysosomal glycolipid transport protein, the GM2-activator protein (GM2AP). The GM2AP extracts a molecule of GM2 from the lysosomal membrane and then the complex specifically binds to soluble Hex A, forming the active quaternary structure. A deficiency of either of the two Hex A subunits or the GM2AP, due to a mutation in their respective genes, can lead to the accumulation of GM2 in the lysosomes of primarily neuronal cells, where the synthesis of the more complex gangliosides is the highest. This storage leads to neuronal cell death and one of three similar neurodegenerative diseases collectively known as GM2 gangliosidosis. These diseases are Tay-Sachs disease (TSD), alpha subunit deficiencies, Sandhoff disease (SD), beta subunit deficiencies and the rare AB-variant form, GM2AP deficiencies. Because of the complexity of assaying Hex activity with its natural substrate (the GM2-GM2AP complex), simple fluorescent artificial substrates were introduced that are hydrolyzed by Hex in a GM2AP-independent manner. The oldest of these is neutral 4-methylumbelliferyl-2-acetamido-2-beta-D- glucopyranoside (MUG). However, when MUG is used to assay total Hex activity in TSD cells, nearly normal enzyme levels are obtained, because of increased levels of Hex B. A newer, more specific, negatively charged version of MUG, 4-methylumbelliferyl-2-acetamido-2-deoxy-beta-D-glucopyranoside-6-sulfate (MUGS) was developed that is only poorly bound and hydrolyzed by Hex B and can thus be used directly to diagnose TSD. In SD both Hex A and B are deficient, but a small amount of Hex activity (∼2% of normal, as measured by MUG) persists due to the inefficient formation of an unstable acidic isozyme, Hex S, an alpha homodimer (alpha monomers are cleared by the endoplasmic reticulum associated degradation system). While human Hex S, like Hex B, is unable to interact with the GM2-GM2AP complex, it can hydrolyze MUGS more efficiently than Hex A. The ∼MUG/MUGS ratios of the Hex isozymes are; Hex A, 4/1; Hex B, 300/1; and Hex S, 1/1 (reviewed in [1]).

The crystal structure of Hex B [2], [3], Hex A [4] and the GM2AP [5] have been elucidated and a model for the active quaternary structure, i.e. Hex A-GM2AP-GM2 complex, generated [2]. Several important observations were made from these structures. Firstly, whereas each subunit has an active site, residues from the neighboring subunit in the dimer are needed to stabilize and complete the site. Thus monomeric subunits are not active. Secondly, the structures confirm previous findings that the ability of the alpha active site to efficiently hydrolyze negatively charged substrates, e.g. MUGS and GM2, comes primarily from two aligned amino acid differences in the subunits, i.e. alpha-N423-R424 and beta-D452-L453. The basic R424 residue in alpha can ion pair with either the 6-sulfate of MUGS [6] or the sialic acid of GM2 [7], whereas the acidic D452 residue in beta repels these same moieties. Finally several areas in both subunits were identified as being potentially important in facilitating the formation of the active quaternary structure. These included two loop structures in alpha, GSEP283 and IPV398. In beta the former alpha-loop sequence aligns with RQNK315, which is removed proteolytically in the lysosome, producing the mature 29 and 24 kDa beta chains [8], [9], and the latter loop is not encoded by the beta mRNA [10]. The importance of the alpha- GESP283, but not the IPV loop in GM2AP-GM2 binding has been confirmed experimentally [11].

Recently Matsuoka et al. used the above information to construct a human hybrid Hex subunit (Table 1) that retained the high stability of the beta subunit while reportedly being able to bind the GM2AP and efficiently hydrolyze GM2 (and MUGS). They suggested that this hybrid Hex could be used for enzyme replacement therapy [12].

**Table 1:** Amino acid changes mage in the H1 and H2 alpha-beta hybrid subunits.

Wild Type beta	H1^1^	H2^1^	Proposed Function
R312	G	G	GM2AP-binding
Q313	S	S	GM2AP-binding
N314	E	E	GM2AP-binding
K315	P	P	GM2AP-binding
L316	No Change	S	GM2AP-binding
D317	No Change	G	GM2AP-binding
T378	No Change	E	GM2AP-binding
D426	No Change	E	Sialic Acid-binding^2^
S427	No Change	DIPV	GM2AP-binding
D452	N	N	Sialic Acid-binding^2^
L453	R	R	Sialic Acid-binding^2^

1 Aligned amino acids from the alpha subunit that were substituted to make the hybrid.

2 Also enhances MUGS binding and hydrolysis.

Another approach to treating TSD and SD is gene therapy. Proof-of-concept gene transfer experiments have demonstrated the potential for long-term therapeutic rescue of GM2 ganglioside accumulations and improvement of disease symptoms in mouse models for SD or TSD [13]–[15]. Adeno-associated virus (AAV) vectors have been utilized in over 75 gene transfer clinical trials because of their excellent safety record, relatively low immunogenicity, and ability to confer long-term expression of the delivered transgene (reviewed in [16]). Recently, widespread central nervous system (CNS) gene transfer has been demonstrated in feline, porcine, and non-human primate animal models [17]–[21], suggesting the possibility for a translatable gene transfer approach for disorders such as Tay-Sachs disease using AAV vectors. A major limitation for AAV is its packaging capacity, which is approximately 4.5 kb of foreign DNA for traditional single-strand AAV, and approximately 2.1 kb for the more efficient self-complementary AAV [16]. The coding DNA sequence for the alpha-subunit of Hex A is 1613 bp, and 1671 bp for the beta-subunit. Packaging the alpha subunit is well within the size constraints of the AAV genome. However, overexpression of the alpha subunit alone would not lead to an overabundance of the missing heterodimeric Hex A isozyme, since the endogenous beta subunit would become limiting in this scenario. Packaging both of these subunits within a single AAV genome, along with the transcriptional regulator elements necessary to drive expression, is impractical due to size constraints. A modified beta subunit that could form a homodimer, but function as an alpha-beta Hex A heterodimer, such as was described by Matsuoka et al. [12], would be of enormous value. Conceptually, such a modified beta subunit gene could be packaged within a self-complementary AAV vector, delivered globally to the CNS, and supply Hex A-like activity to the majority of cells in the CNS through cross-correction (secretion of the overexpressed homodimer from infected cells, and recapture of the isozyme by plasma membrane mannose-6-phosphate receptors on uninfected cells) [22].

Given the promising data published by Matsuoka et al. [12] and our interest in developing gene therapy for TSD and SD utilizing a single AAV vector, we decided to confirm and build on their previous work. This included constructing an identical Hex hybrid (Hex H1) and comparing its properties to a new hybrid (Hex H2) containing additional alpha-sequences that our active quaternary structure model [2] predicted to be involved in GM2AP or sialic acid binding (Table 1). These hybrids were then tested in cellulo (live cell-based) with our previously validated, GM2AP-dependent, assay utilizing a fluorescent GM2 derivative, i.e. 2-nitro1,3-benzoxadiazol (NBD)-4-yl, covalently attached to a short (C6) sn2 acyl chain of lyso-GM2 ganglioside (NBD-GM2), as the substrate [23] and in vitro after ion-exchange separation of the Hex isozymes, with and without the addition of recombinant human His6-tagged wild-type (WT) GM2AP (rGM2AP) [24].

## Results and Discussion

**Figure 1:**
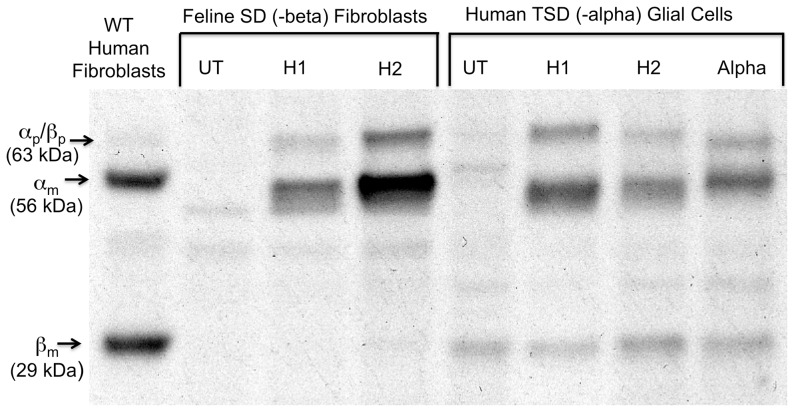
Western blot analysis of the lysate from wild-type (WT) human fibroblasts (positive control), untransfected (UT) Feline SD fibroblasts and human TSD Glial cells (negative control), and a clonal population of the indicated cell types highly expressing either the H1 or H2 beta-alpha hybrid subunit (**Table 1**) or the WT alpha subunit. The positions of the alpha (α_p_) and beta (β_p_) precursor polypeptides and the mature (lysosomal) alpha (α_m_) and beta (β_m_) chains are indicated. Note that both hybrids lack the internal posttranslational cleavage site present in the beta, but not the alpha subunit, that has been linked to GM2AP binding.

Expression vectors encoding the H1 and H2 Hex hybrids were individually transfected into both an immortalized feline SD fibroblast line (homozygous for a 25 bp inversion at the 3′ end of the beta-coding sequence [25], an area involved in dimerization [2]) and a human infantile TSD Glial cell line [26], and transiently expressed to confirm that their expression would result in increased levels of MUGS hydrolysis (Table 2). Some TSD cells were also transfected with a vector encoding the human WT alpha subunit as a positive control. Initial MUGS levels were clearly above those of the untransfected (UT) cells, but were too low to obtain conclusive results using either our in cellulo or in vitro NBD-GM2 assays [23]. Selection of mixed colonies of permanently transfected cells increased the specific activity of MUGS hydrolysis by only 2–3 fold, which was still insufficient for the NBD-GM2 assay. Thus, individual clonal populations of the permanent lines were isolated, grown and assayed in order to identify those clones that were expressing high levels of MUGS activity (Table 3). The specific MUGS activities of ∼25 clonal lines stably expressing each construct, i.e. 125 clones, were assessed and 12 high-expressing clonal lines selected (Table 3) for growth and expansion. From these 12 lines a single H1 and alpha high-expressing, clonal TSD Glial line, as well as two clonal lines expressing H2 (at either a high or moderately high level) were grown for in cellulo cell-feeding assays with NBD-GM2 (Table 4). Similarly the two clonal feline SD fibroblast lines that highly expressed either H1 or H2 were established and grown for cell-feeding assays (Table 3 and 4). The expression of H1, H2 and alpha was confirmed in the high-expression clonal cell lines by Western blot analysis using a human specific rabbit IgG against Hex A [27], which cross-reacts with both the human alpha and beta subunits, but not the SD feline alpha subunit (Fig. 1). All of the above data confirm that, as previously reported [6], the substrate specificity of the beta subunit-based hybrids had indeed been changed so that they efficiently hydrolyzed negatively charged MUGS and potentially GM2 (Table 3).

**Table 2:** Transiently Transfected Cells and Selected Mixed Colonies.

Cells	UT^1^	Alpha	H1	H2
Human TSD Glial^2^	1.3^3^	56^3^	22^3^	13^3^
Feline SD Fibroblasts^2^	7.7	NA^4^	34	23
Human TSD Glial^5^	1.7	110	46	36

1 Untransfected.

2 Transiently transfected.

3 Specific activity of the cell lysates, nmoles (MUGS) hr^−1^ mg^−1^ (total protein), note that the specific activity of WT human fibroblast lysate is 220.

4 Not Applicable.

5 G418 selected mixed colonies.

**Table 3:** Stably Transfected, Highly Expressing Clones from Selected Mixed Colonies.

Cells	UT^1^	Alpha	H1	H2
Human TSD Glial	3.2^2^			
Clone-1^3^		421^2^	409	270
Clone-2		2090	1430	152
Clone-3		750		435
Clone-4		1210		
Clone-5		385		
Feline SD fibroblasts	5.1			
Clone-1		NA^4^	272	762

1 Untransfected.

2 Specific activity of the cell lysates, nmoles (MUGS) hr^−1^ mg^−1^ (total protein).

3 Highly expressing clones isolated.

4 Not Applicable.

**Table 4:** Cell Feeding Experiment: Total amounts of MUGS Units (nmoles/hr) and protein (mg) in each sample extracted for HPTLC analysis.

Cell Type	UT^1^			H1			H2			Alpha		
Human TSD Glial (Units)	10	6.5	7.2	350^2^	230^2^	200^2^	97^3^	540^2^	540^2^	860^2^	1200^2^	1000^2^
Human TSD Glial (mg)	2.4	1.9	1.7	0.8	0.6	0.5	1.9	1.2	1.5	0.8	1.5	0.9
Feline SD Fibroblasts (Units)	9.7	10	8.3	180^2,4^	240^2^	230^2^	1300^2,4^	1300^2^	1400^2^	NA^5^	NA	NA
Feline SD Fibroblasts (mg)	0.9	1.1	1.1	0.7	0.9	0.9	0.9	1.2	1.3	NA	NA	NA

1 Untransfected.

2 High expressing clone (single clone used for each of these determinations).

3 Moderately high expressing clone.

4 ∼25% of the MUGS units in these samples were found to be associated with alpha-H1 or alpha-H2 heterodimers (Fig. 4).

5 Not Applicable.

**Figure 2:**
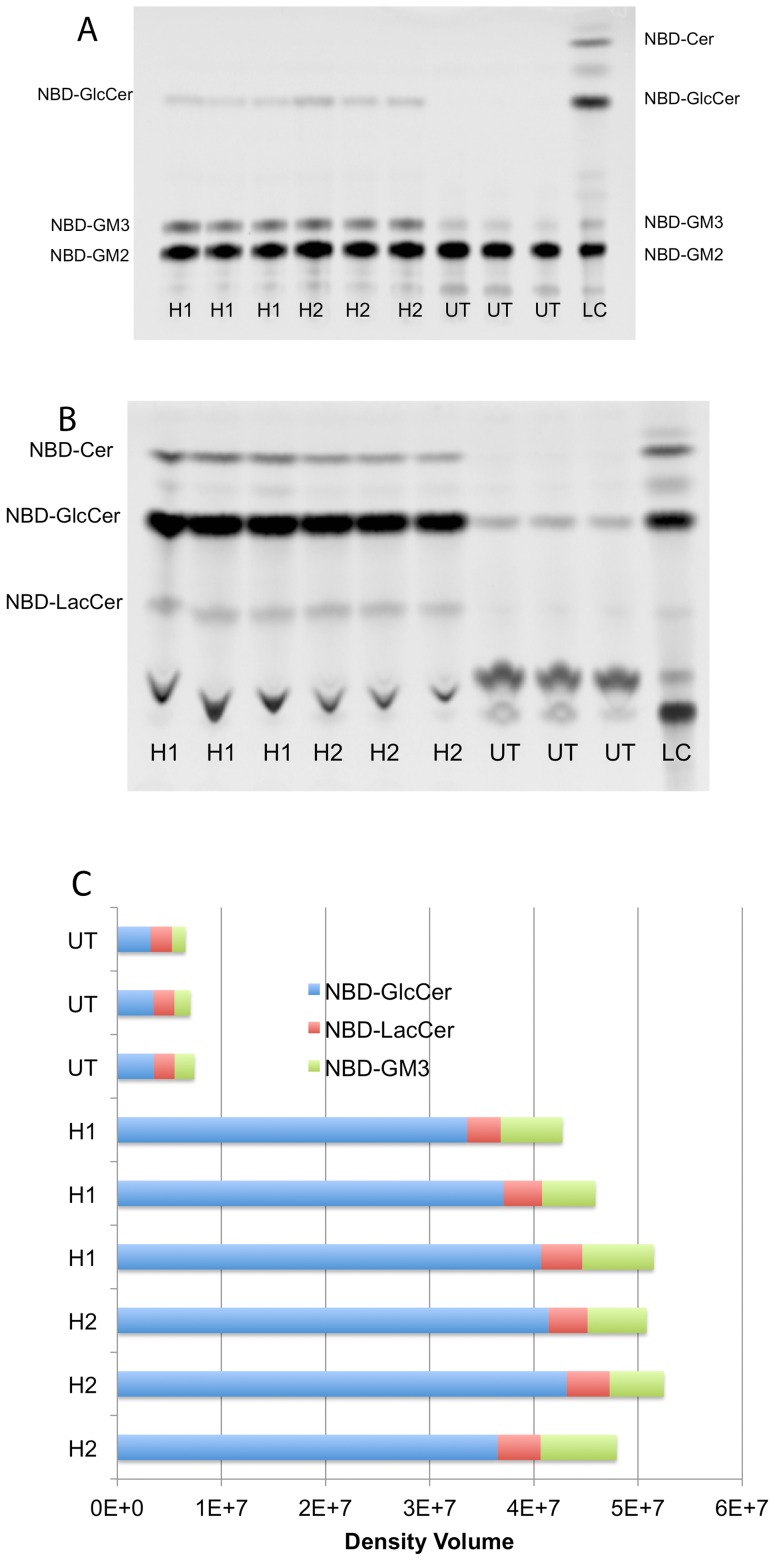
In cellulo NDB-GM2 hydrolysis assays of nine independently treated plates of three sets of Feline SD fibroblasts. The three sets include, isolated clonal populations of highly expressing H1 or H2 beta-alpha hybrid subunit and untransfected (UT) controls. See Table 4 for total MUGS units and cell protein contained in the cell lysates that were examined in these assays. Loading control (LC) shows the positions of the substrate NBD-GM2, and it break-down products; i.e, NBD-GM3, NBD derivative of GM3 ganglioside; NBD-LacCer, NBD derivative of lactosylceramide; NBD-GlcCer, NBD derivative of glcosylceramide; NBD-Cer, NBD derivative of ceramide (note that only low levels of NBD-Cer are seen because of the presence of CBE in the assay, see methods). A) The HPTLC separation of the enriched acidic glycolipid fraction (upper phase Folch extraction) from each plate of cells. B) The HPTLC separation of the enriched neutral glycolipid fraction (lower phase Folch extraction) from each plate of cells. C) Stacked bar graph of the NBD-GM2 hydrolysis products detected in A & B quantified using the Storm Imager.

Three replicate plates of transfected (Hex H1 and H2) and UT feline SD fibroblasts were grown in the presence of NBD-GM2 and conduritol-B-epoxide (CBE), which is an irreversible inhibitor of glucocerebrosidase [28] and stops the degradation of NBD-GM2 in lysosomes at the NBD derivative of glucosylceramide step. Thus, as well as the acidic glycolipid product, NBD-GM3 ganglioside, the reaction also produces two neutral glycolipid products, a NBD-lactosylceramide derivative and NBD-glucosylceramide [23] (Fig. 2 A & B). Cells from the 9 independent plates were harvested and 10% of each cell suspension was removed for MUGS and protein assays (Table 4). Enriched neutral and acidic glycolipid fractions were prepared from the remaining 90% of treated cells by Folch extraction, and individually analyzed by HPTLC. Cells expressing either of the H1 or H2 hybrid clearly were able to hydrolyze more of the NBD-GM2 than UT cells (Fig. 2A, B, C).

**Figure 3:**
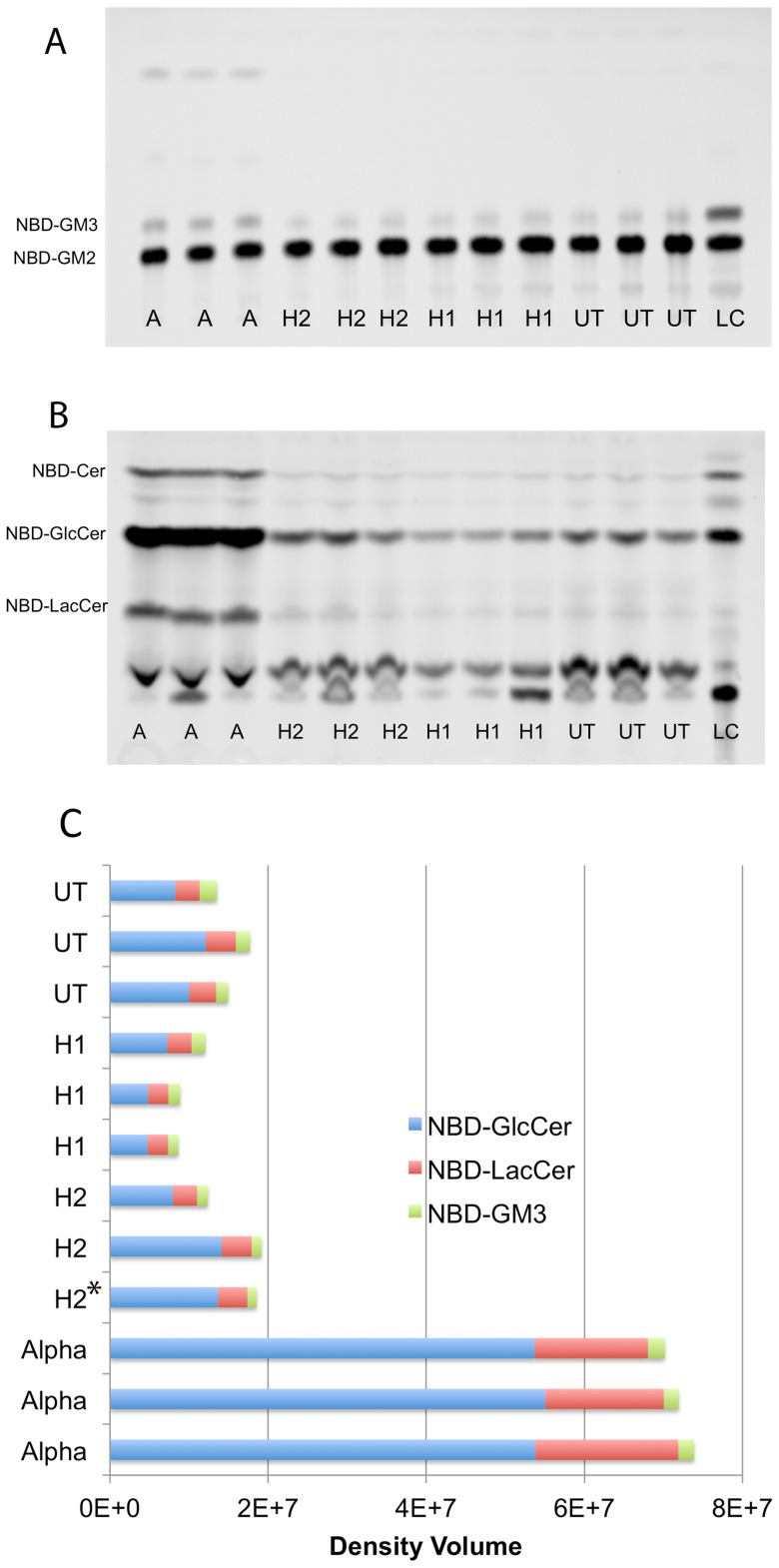
In cellulo NDB-GM2 assays of twelve independent treated plates of four sets of human TSD Glial cells. These sets include, isolated clonal populations highly expressing either the WT alpha subunit (A), the H2 or the H1 beta-alpha hybrid subunit and untransfected (UT) controls (see Table 4 for total MUGS units and cell protein examined in these assays). Loading control (LC) shows the positions of the substrate, NBD-GM2, and its break-down produces; i.e, NBD-GM3, NBD derivative of GM3 ganglioside; NBD-LacCer, NBD derivative of lactosylceramide; NBD-GlcCer, NBD derivative of glcosylceramide; NBD-Cer, NBD derivative of ceramide (note that only low levels of NBD-Cer are seen because of the presence of CBE in the assay, see methods). A) The HPTLC separation of the enriched acidic glycolipid fraction (upper phase Folch extraction) from each plate of cells. B) The HPTLC separation of the enriched neutral glycolipid fraction (lower phase Folch extraction) from each plate of cells. C) Stacked bar graph of the NBD-GM2 hydrolysis products detected in A & B (H2* indicates the moderately high expressing clone, see Table 4) quantified using the Storm Imager.

Similar in cellulo assays were done with 12 independent plates of 4 clonal populations of transfected human TSD Glial; i.e., high expressing H1 (three replicate plates), H2 (two replicate plates), WT alpha subunit (three replicate plates), and a moderately high expressing H2 subunit (one plate); and three replicate plates of UT control cells (Table 4, Fig. 3A, B, C). Surprisingly only the positive control cells transfected with the human WT alpha cDNA hydrolyzed significantly more NBD-GM2 than did the UT negative control cells (Fig. 3C).

**Figure 4:**
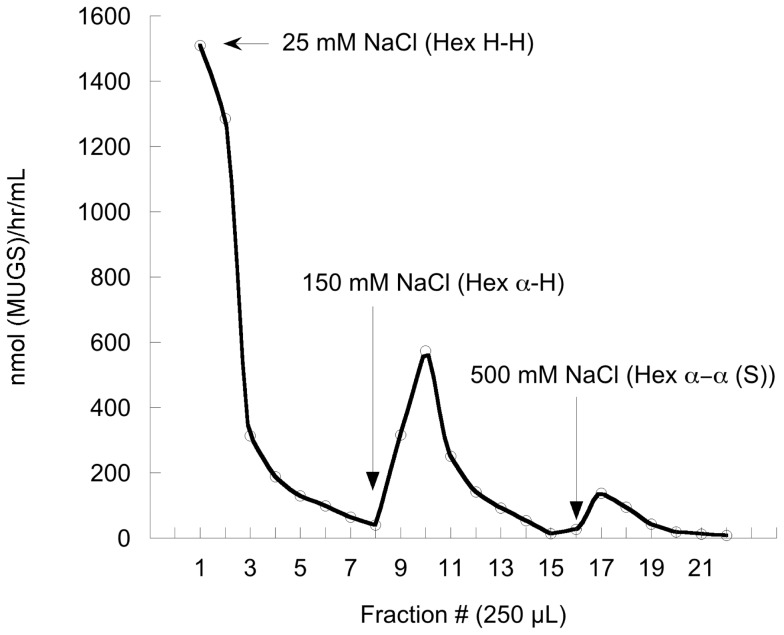
DEAE ion-exchange separation of the Hex isozymes from the lysate of transfected feline SD fibroblasts highly expressing the H1 beta-alpha hybrid (similar results were obtained using lysate from these cells expressing the H2 hybrid, thus a generic “H” is used for the hybrid subunit).

**Figure 5:**
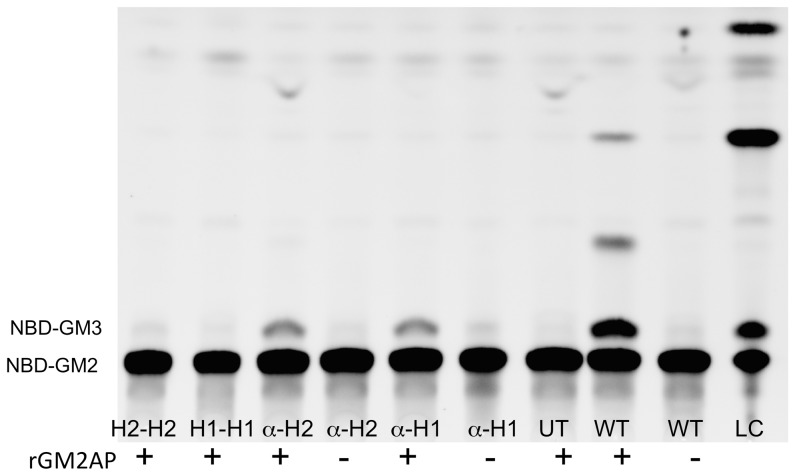
HPTLC separation of the total glycolipid fraction from an in vitro assay (NBD-GM2± rGM2AP) of the DEAE-separated Hex isozymes (see Fig. 4) from lysates of transfected feline SD fibroblasts highly expressing either the H1 or H2 beta-alpha hybrid subunits. Lysate from normal fibroblasts, containing the same number of MUGS units as the test samples (15 nmoles/hr), was used as the positive control (WT). Lysate from untransfected feline SD cells, containing the same amount of protein as the WT lysate, was used as the negative control (UT).

The most likely explanation for the different results obtained from the SD versus the TSD cell lines is that the beta subunit-based hybrids were able to form homodimers (which could now hydrolyze MUGS, but could still not interact with the GM2AP-NBD-GM2 complex) and heterodimers with the different endogenous Hex subunits present in the two transfected cell lines. In the feline SD cells any hybrid heterodimer would contain an endogenous feline alpha subunit, producing a Hex A-like isozyme (alpha-hybrid) that may then be able to interact with the feline GM2AP and hydrolyzed NBD-GM2. In the human TSD cells, any hybrid heterodimer would contain an endogenous human beta subunit, producing a Hex B-like isozymes (beta-hybrid) that now could hydrolyze MUGS, but may still not be able to interact with the human GM2AP-NBD-GM2 complex. To test this hypothesis we took advantage of the facts that both hybrids retained approximately the same neutral pI as the WT human beta subunit and that the feline alpha-subunit has the same acidic pI as its human counterpart. Thus hybrid homodimers could be separated from any alpha (WT feline)-hybrid (human beta subunit-based) heterodimers by standard DEAE ion exchange chromatography (reviewed in [1]). At pH 6.0 and 25 mM NaCl (10 mM phosphate buffer) Hex B (and either beta subunit-based hybrid homodimer) does not bind to the column, Hex A (and any feline alpha-human beta subunit-based hybrid heterodimer) is eluted at 150 mM NaCl and Hex S at 500 mM NaCl [11]. A stepwise elution of the ion exchange column loaded with lysates of feline SD cells transfected with either hybrid construct, produced a large peak of MUGS activity that did not bind to the column, which signified the beta-subunit based hybrid homodimer. Additionally a smaller peak of MUGS activity was eluted with 150 mM NaCl, confirming the presence of alpha-hybrid heterodimers (accounting for ∼25% of the cells’ MUGS activity). Finally a very small Hex S peak was eluted with 500 mM NaCl (Fig. 4). The two peak fractions eluted by either 25 mM or 150 mM NaCl were individually pooled and 15 nmoles (MUGS)/hr of Hex activity from each were assayed in vitro with NBD-GM2 contained in negatively charged liposomes, with or without added rGM2AP [23]. As there is little additional turn-over of the NBD-GM3 product in the in vitro assay [23] the total glycolipids were bound to, washed and eluted (methanol) from a C-18 Zip tip, instead of using a differential Folch extraction [29] as was done in the in cellulo assays (above). These data confirmed that the alpha- H1 and -H2 heterodimers, but not the H1-H1 and H2-H2 homodimers, are capable of hydrolyzing NBD-GM2 to produce NBD-GM3, in a rGM2AP-dependent manner (Fig. 5).

Recently Matsuoka et al. reported that they had successfully constructed a hybrid Hex subunit (H1) that as a homodimer retained the high stability of Hex B and also was able to bind the GM2AP-GM2 complex and hydrolyze GM2 [12]. This conclusion was based on three experiments. The first was the demonstration that their Hex H1 was able to hydrolyze GM2 in the presence of 2mM taurodeoxycholate. The substitution of GM2AP with this detergent circumvents the need to form the active quaternary structure [30], which is used in vivo. In this case the experiment confirms that the Hex H1 is able to hydrolyze negatively charged substrates (MUGS and GM2), but did not test the isozyme’s ability to bind the GM2AP-GM2 complex. The second experiment demonstrated that recombinant hybrid Hex injected intracerebroventricularly into SD mice was therapeutic, lowering the amounts of stored ganglioside. One of the major problems in translating data from mouse models of TSD and SD to humans is the presence in mice of a metabolic bypass pathway for GM2 hydrolysis not found in humans. This pathway consists of a mouse sialidase, which can convert GM2 to its neutral, asialo derivative GA2 (now a potential substrate for the beta active site), coupled with a more promiscuous mouse GM2AP, which unlike the human GM2AP, can both weakly bind GA2 and interact with Hex B [31]. Thus the TSD mouse is only very mildly affected as compared to either the SD mouse [32], [33] or the GM2AP-deficient AB-variant mouse [34]. Since the mouse GM2AP can interact with mouse (or human) Hex B [31], [35], it is very likely to also be able to interact with human Hex H1. Additionally, because Hex H1 can hydrolyze negatively charged substrates, the need for mouse sialidase to first convert GM2 to GA2 is eliminated, making the mouse bypass pathway more efficient. The third experiment; demonstrating that endogenous GM2 levels in human TSD fibroblasts could be reduced by supplying Hex H1 in the growth medium; is more difficult to explain. However, this conclusion was based only on immunostaining of the cells with an anti-GM2 mouse monoclonal antibody viewed under a confocal fluorescent microscope. Problems with this approach could relate to the specificity and/or sensitivity of the antibody, since there are only low levels of GM2 and the other complex gangliosides synthesized outside of neuronal cells. Thus, while fibroblasts from GM1 or GM2 gangliosidosis patients may contain elevated levels of GM1 or GM2, these levels remain very low and difficult to quantitate [36]–[38], i.e. fibroblasts are not a storage cell-type in TSD. For this reason, Igdoura et al. used TSD neuroglia cells to examine changes in endogenous GM2 turnover, but when they evaluated TSD fibroblasts they first preloaded the cells with exogenous GM2 [37]. To validate the specificity of their GM2 antibody and the presence of stored GM2 in TSD neuroglia cells, the same group first extracted the glycolipids from the cells and then separated them by HPTLC. The HPTLC plate was then overlaid with their GM2 antibody and visualized in a manner similar to a Western blot [33]. This type of experiment should always be done as a control for the ability of GM2 antibody being used to specifically detect stored GM2 in the TSD or SD cell-type being treated.

In conclusion, constructing a modified beta subunit that can form a homodimer and, like heterodimeric Hex A, hydrolyze GM2 in vivo, would be of great value in developing gene therapy for TSD and SD utilizing a single AAV vector. While identifying the changes to the beta active site necessary for it to efficiently bind and hydrolyze a simple negatively charged substrate, MUGS, has been accomplished [2], [6], [12], the remaining hurdle is to identify all the alpha residues that need to be incorporated into the beta hybrid to allow it to effectively bind the human GM2-GM2AP complex. This report demonstrates that this hurdle has yet to be jumped.

## Materials and Methods

### Plasmid Constructs

The Hex alpha subunit and modified Hex beta subunits were codon-optimized for mouse and human expression by DNA2.0 (Menlo Park, CA). The coding DNA sequences were cloned into the pJ603 mammalian expression vector (DNA2.0), which drives the Hex subunit expression via the CMV promoter and also co-expresses the neomycin resistance gene. The entire alpha subunit coding sequence was cloned into the expression construct. For the modified beta subunits, the coding sequence beginning at the 3^rd^ ATG was cloned into the expression construct to minimize the size of the coding sequence (deleting the first 25 amino acids of the long, cleavable signal peptide), since this N-terminus was previously shown to be dispensable [39].

### Cell Lines and Tissue Culture

Immortalized fibroblast cell lines from a naturally-occurring feline SD model (GM2 SV3) were generated by D.R. Martin [25] by calcium phosphate transfection of primary fibroblasts with the plasmid pSV3-DHFR (American Type Culture Collection) containing the large T antigen of Simian Virus 40. An immortalized human Tay-Sachs Glial cell line was obtained from R.A. Gravel [26]. All cells were grown in alpha-minimal essential medium from Wisent Inc. (Canada) in the presence of 1% antibiotics (penicillin and streptomycin, Gibco BRL, Canada) and supplemented with Fetal Bovine Serum (FBS) (Wisent Inc., Canada) at 10% for human cells or 1% for cat cells, and incubated at 37°C in a humidified atmosphere with 5% CO_2_.

### Chemicals and Hex Assays

The synthetic fluorogenic substrate, MUGS, from Toronto Research Chemicals (Canada), was used to assay Hex A-like activity (e.g. Hex S and the hybrids) as previously reported [40]. CBE was from Toronto Research Chemicals (Canada). Cholesterol, purchased from Sigma-Aldrich (Canada), phosphatidyl choline (egg) and phosphatidyl inositol (bovine liver) from Avanti Polar Lipids (USA), and polycarbonate 100 nm filters from Avestin, Inc. (Canada), were used to produce the previously described [23] negatively-charged liposomes that the NBD-GM2 substrate was incorporated into for the in vitro Hex assays (see below). Recombinant GM2AP was expressed in *Escherichia coli* then purified (His6-tagged) and re-folded [24].

#### In cellulo NBD-GM2 assay

The fluorescent GM2 derivative, NBD-GM2, was prepared by W. Wakarchuk [23]. Confluent transfected or non-transfected cells in 10 cm plates were grown for 18 h in FBS-free media containing NBD-GM2 (4.7 µg mL^−1^) and CBE (50 µM). After media removal, the cells were rinsed with PBS and incubated with media containing 5% FBS for an additional 2 h before harvesting. The differential extraction of gangliosides and neutral glycolipids from each cell suspension was done according to Folch [29]. The extracts were then cleaned using C-18 Zip Tips and prepared for glycolipid separation by HPTLC as previously reported [40]. Bands corresponding to NBD-glycolipid derivatives were visualized and quantified using the Storm Imager [40].

#### In vitro NBD-GM2 assay

Aliquots containing 15 nmoles (MUGS)/hr of Hex activity from the DEAE ion-exchange separation of the Hex isozymes (see below), were incubated overnight in McIlvaine’s citrate phosphate buffer (pH 4.1), with NBD-GM2 incorporated into negatively-charged liposomes and 50 µM CBE plus or minus 2 µg rGM2AP, in a total reaction volume of 50 µL. The glycolipids (both acidic and neutral) were bound in a C-18 Zip tip, washed with water, eluted with 100% methanol and concentrated by drying before their separation by HPTLC, as previously reported [40].

### Protein Analyses

#### Western blotting

Lysates (20 µg of total protein) from human WT fibroblasts, feline SD fibroblasts and human TSD Glial cells were subjected to SDS-PAGE on a 10% bis:acrylamide gel, transferred to nitrocellulose, and processed as previously described [27]. Blots were incubated with a rabbit polyclonal IgG produced in our laboratory against purified human Hex A [27], followed by a horseradish peroxidase-conjugated, goat, anti-rabbit IgG secondary antibody, developed using chemiluminescent substrate according to the manufacturer’s protocol (Amersham Biosciences, UK) and recorded on BIOMAX x-ray film (Kodak) [40].

#### Ion-exchange chromatography

DEAE Sepharose CL-6B (Pharmacia), 250 µL, was pre-equilibrated in a small column with10 mM phosphate buffer pH 6.0 containing 25 mM NaCl and 5% glycerol. Cells from two 15 cm plates were harvested and lysed by repeated freeze- thawing in the above 10 mM phosphate buffer. The lysates, 500 µL, were clarified by centrifugation, passed through individual DEAE columns and collected as the first fraction shown in Figure 4. Each column was washed with a further 1.5 mL, collected in 6, 250 µL fractions. Finally 1 mL of the lysis buffer was used as a final wash and collected as a single fraction. Only the two fractions containing the highest MUGS activity were pooled and used to evaluate the ability of the Hex hybrid homodimers to hydrolyze NBD-GM2 in a rGM2AP-dependent manner in vitro. Each column was then eluted with 1.5 mL of the phosphate buffer containing 150 mM NaCl, collected in 6, 250 µL fraction, followed by another 1 mL wash with the same buffer. The two fractions with the highest levels of MUGS activity were again pooled as alpha-hybrid heterodimers and assayed in vitro with rGM2AP and NBD-GM2. Finally the columns were eluted with 1.25 mL of buffer containing 500 mM NaCl to collect 5, 250 µL fractions containing the alpha homodimers, Hex S, followed by a final 1 mL wash. All the fractions were assayed with MUGS (Fig. 4).
